# Comparative Analysis of the Metabolic Profiles of Strains of *Tribolium castaneum* (Herbst) Adults with Different Levels of Phosphine Resistance Based on Direct Immersion Solid-Phase Microextraction and Gas Chromatography-Mass Spectrometry

**DOI:** 10.3390/molecules28237721

**Published:** 2023-11-22

**Authors:** Li Li, Changyao Shan, Qun Liu, Baishu Li, Tao Liu

**Affiliations:** Institute of Equipment Technology, Chinese Academy of Inspection and Quarantine, No. A3 Gaobeidianbeilu, Chaoyang District, Beijing 100123, China; lili@caiq.org.cn (L.L.); 34424808@student.murdoch.edu.au (C.S.); liuq@caiq.org.cn (Q.L.); libaishu@163.com (B.L.)

**Keywords:** *Tribolium castaneum* (Herbst), DI-SPME-GC-MS, metabolites, PH_3_ resistance detection, pest management

## Abstract

The management of phosphine (PH3) resistance in stored grain pests is an essential component of implementing timely and effective pest control strategies. The prevailing standard method for PH3 resistance testing involves the exposure of adult insects to a specific concentration over a fixed period. Although it is widely adopted, this method necessitates an extensive period for assay preparation and diagnosis. To address this issue, this study employed Direct Immersion Solid-Phase Microextraction (DI-SPME) coupled with Gas Chromatography-Mass Spectrometry (GC-MS) to compare and analyze the metabolic profiles of PH3-sensitive (TC-S), PH3 weak-resistant (TC-W), and PH3 strong-resistant (TC-SR) *Tribolium castaneum* (Herbst) adults. A total of 36 metabolites were identified from 3 different PH3-resistant strains of *T. castaneum*; 29 metabolites were found to present significant differences (*p* < 0.05) across these groups, with hydrocarbon and aromatic compounds being particularly prevalent. Seven metabolites showed no significant variations among the strains, consisting of four hydrocarbon compounds, two iodo-hydrocarbon compounds, and one alcohol compound. Further multivariate statistical analysis revealed a total of three, two, and nine differentially regulated metabolites between the TC-S versus TC-W, TC-S versus TC-SR, and TC-W versus TC-SR groups, respectively. Primarily, these metabolites comprised hydrocarbons and iodo-hydrocarbons, with the majority being associated with insect cuticle metabolism. This study demonstrates that DI-SPME technology is an effective method for studying differentially expressed metabolites in *T. castaneum* with different levels of PH3 resistance. This approach may help to provide a better understanding of the development of insect PH3 resistance and act as a valuable reference for the establishment of rapid diagnostic techniques for insect PH3 resistance.

## 1. Introduction

*Tribolium castaneum* (Herbst), commonly known as the red flour beetle, is a global stored grain pest. It has the potential to inflict damage on a wide array of commodities, more than 20%, thereby causing substantial economic losses, particularly in developing countries [1]. Over the past 50 years, PH_3_ has emerged as a preferred fumigant due to its cost-effectiveness, rapid diffusion, and low residual levels. However, the long-term irrational use of this single chemical increases the risk of PH_3_ resistance among *T. castaneum* populations [2]. Recent reports show that key grain-producing regions, including Asia, North America, South America, Europe, and Africa, have alarming levels of PH_3_ resistance [3,4]. This finding not only highlights the gravity of the situation but also focuses on the immediate necessity to address this urgent problem. Consequently, the grain industry is now confronted with an urgent demand for precise and rapid methods to detect PH_3_ resistance in insects [5,6]. These methods are essential for designing rational fumigation strategies, thereby offering innovative approaches and solutions to ensure food security and effective pest management.

The method established by the Food and Agriculture Organization (FAO) in 1975 serves as the standard procedure for diagnosing resistance to PH_3_ in pest populations. The diagnostic process involves two stages of mortality evaluations following the bioassay. The first stage is an initial assessment conducted at the conclusion of the fumigation phase, and the second stage is a subsequent confirmatory assessment of endpoint mortality 14 days after the fumigation period has ended [4]. The main limitation of this bioassay method, which is based on mortality rate, is the excessive time required for resistance diagnosis. The Knockdown Test (KT) method, a variation of the FAO method, offers a rapid assay for PH_3_ resistance based on time to knockdown. This assay relies on the response variable “behavior,” which is defined as the inability of an insect to walk in a coordinated manner [7,8,9]. However, the ambiguity of the KT discriminant criteria may lead to significant variations among the results obtained by different testers, thereby causing the diagnostic outcomes to potentially deviate substantially from the actual levels of resistance. Molecular resistance diagnosis is emerging as an alternative to the traditional FAO bioassay [10,11], but this technique requires a relatively high level of initial financial investment and specialized knowledge.

The toxicological mechanism of PH_3_ involves disrupting the sympathetic nervous system, inhibiting energy metabolism, and altering cellular redox balance [12]. Two mitochondrial enzymes—*cytochrome b5 fatty acid desaturase* (*Cyt-b5-r*) and *dihydrolipoamide dehydrogenase* (*DLD*)—have been defined as *rph1* and *rph2*, key factors in PH_3_ resistance in various organisms, including several stored grain pests. However, the molecular mechanisms of these two key genes involved in PH_3_ toxicity remain unclear [13]. Recent studies suggest that variations in the levels of certain metabolites within insects significantly affect pesticide resistance, providing a new perspective on resistance mechanisms [14]. Specifically, compounds such as lipids and hydrocarbons appear to form a protective barrier that reduces insect susceptibility to PH_3_ [15,16,17,18]. Consequently, changes in specific insect metabolites may serve as biomarkers that indicate the level of PH_3_ resistance.

Solid-Phase Microextraction (SPME) has been widely recognized as a rapid sample processing technique, especially in the fields of analytical biology, pharmaceuticals, and food research. Compared to traditional detection methods, SPME coupled with GC-MS enhances the purity, reproducibility, and sensitivity of the extracts [19]. However, its primary limitation stems from its interest in volatile and semi-volatile compounds. While the majority of studies concerning insect volatile substances have concentrated on their roles in attraction or repellent effects (i.e., insect pheromones) [20,21], several innovative findings have been reported. For instance, Du et al. utilized HS-SPME to identify two benzoquinone derivatives with potential as biomarkers for detecting *T. castaneum* PH_3_ resistance [14]. Al-Khshemawee et al. employed DI-SPME chemical extraction technology coupled with GCMS to analyze metabolic changes in *Ceratitis capitata* (Wiedemann) during mating [22]. Furthermore, Alnajim et al. extracted and analyzed hydrocarbons in the cuticle of PH_3_-resistant and sensitive strains of *T. castaneum* and *Rhyzopertha dominica* (Fabricius) using DI-SPME-GC-MS, revealing that the high content of hydrocarbons might serve as a key factor in preventing PH_3_ entry into resistant insect bodies [16,17]. These studies demonstrate the high applicability of SPME in the field of insect PH_3_ resistance detection, facilitating not only the rapid diagnosis of population resistance to PH_3_ but also the exploration of potential PH_3_ resistance mechanisms. These insights hold practical implications for PH_3_ resistance management. Additionally, this technology affords researchers the ability to diagnose the PH_3_ resistance level of field-collected insect populations within the same day, thereby facilitating the timely implementation of specific management strategies.

**Figure 1:**
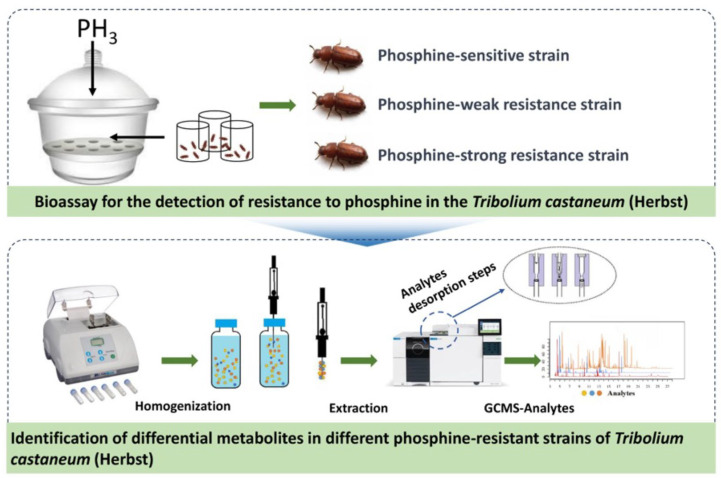
Schematic diagram of the technical process used to explore the metabolic changes in *T. castaneum* strains with different levels of PH_3_ resistance via DI-SPME coupled with GC-MS.

Recognizing the substantial potential of Solid-Phase Microextraction (SPME) technology for differentiating levels of PH_3_ resistance in insects, for this study, we employed DI-SPME to investigate the dynamic changes of volatile organic metabolites within the *T. castaneum* across different PH_3_ resistance levels. This leads to the development of a PH_3_ resistance identification model. Figure 1 illustrates the processes we adopted to achieve the above purposes: First, the strains of *T. castaneum* samples that are sensitive, weakly resistant, and strongly resistant to PH_3_ are selected using the FAO method. Subsequently, DI-SPME-GCMS technology is utilized to extract and characterize the metabolites present in these three strains. Further, based on GC-MS analysis, metabolic profiles are constructed for the samples, and through the application of multivariate statistical analysis methods, a PH_3_ resistance identification model is formulated, identifying the metabolites that are differentially regulated under varying PH_3_ resistances. In addition, to explore the metabolic pathways of PH_3_ resistance, we individually evaluated key differential metabolites. This analysis holds long-term theoretical significance for understanding the underlying mechanisms of PH_3_ resistance in *T. castaneum*.

## 2. Results and Discussion

### 2.1. Phosphine Susceptibility Tests

Probit analysis was conducted on the adult mortality rates of three *T. castaneum* strains (Wuhan, Qihe and Zibo), revealing significant alignment (*p* < 0.001) with complementary log-log and probit-log regression models in response to varying PH_3_ concentrations. These strains showed less variability (heterogeneity < 1), indicating that mortality response to PH_3_ was concentrated. The G-factor, a potency estimation index for mortality response data, ranged from 0.027 to 0.284, maintaining accurate lethal concentration predictions at various probability levels. The G-factor of the three *T. castaneum* strains was below the threshold of 0.5 and ranged between 0.027 and 0.284. This suggests that, based on the G-factor, the predicted lethal concentration ranges at various probability levels are reliable.

Resistance ratios were typically calculated at the 50% mortality level using the FAO method. The LC_50_ for the susceptible strain exposed for 20 h was 0.0088 mg/L (95% fiducial limits: 0.0085–0.0090 mg/L). In accordance with the literature, the adults of *T. castaneum* were classified into three different groups based on their levels of resistance to PH_3_: susceptibility, weak resistance (1 < RR < 100), and strong resistance (RR > 100) [23]. Therefore, in this experiment, the Wuhan, Qihe, and Zibo strains exhibit susceptibility, weak resistance, and strong resistance, respectively (Table 1).

### 2.2. Metabolite Expression in Response to Tribolium Castaneum (Herbst) Adults of Different Phosphine Resistance Levels

**Figure 2:**
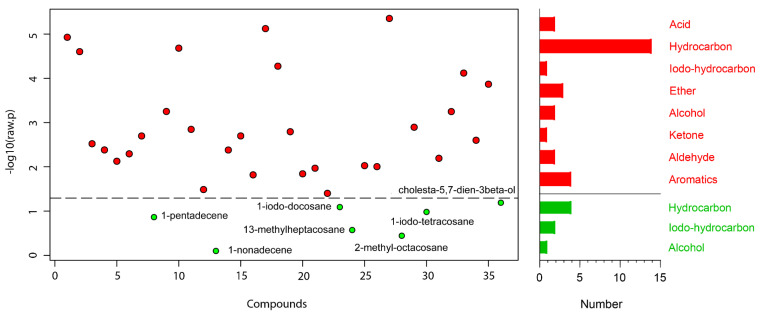
Metabolites obtained in *T. castaneum* adults with different levels of PH_3_ resistance. The 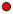 represent significant compounds selected based on the *p*-value threshold (<0.05), and the 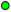 represent non-significant compounds (**left**). The number of metabolites in each chemical classification (**right**).

A total of 36 compounds were identified from 3 strains: 29 from TC-S, 36 from TC-W, and 32 from TC-SR (Table 2). While some compounds were ubiquitous across all three strains, others demonstrated differential expression in one or two strains. A one-way ANOVA and subsequent post hoc analysis (Fisher‘s LSD) revealed significant differences (*p* < 0.05) in the GC-MS response of 29 compounds, including 14 hydrocarbons; 4 aromatics; 3 ethers; 2 each of acids, alcohols, and aldehydes; and 1 each of iodo-hydrocarbons and ketone (Figure 2). Compounds such as 2-methyl-*p*-benzoquinone; (*E*)-2-decenal; 2-undecenal; (*Z*,*Z*)-1,8,11-heptadecatriene; 1,13-tetradecadiene; *n*-hexadecanoic acid; tetracosane; 13-methylheptacosane; 11-methylpentacosane; 2-methylheptacosane; 2-methyloctacosane; docosyl heptyl ether; hentriacontane; and cholesta-5,7-dien-3beta-ol were found to be upregulated with the increase in PH_3_ resistance level in *T. castaneum*. Conversely, compounds such as orcinol; ethyl-*p*-hydroxybenzoate; (*Z*)-8-dodecenol; 1-iodo-tetracosane; 1-iodo-docosane; and 1-iodo-hexacosane were downregulated as the resistance to PH_3_ in *T. castaneum* increased. The relative abundance (RA) of 1-pentadecene remained consistent and was found in the highest proportions among the *T. castaneum* strains with varying levels of PH_3_ resistance. This could be attributed to the distinctive role of 1-pentadecene as a characteristic volatile compound produced by *T. castaneum* [24,25]. Representative chromatograms and mass spectra for each group are provided in the Supplementary Materials (Figure S1).

**Figure 3:**
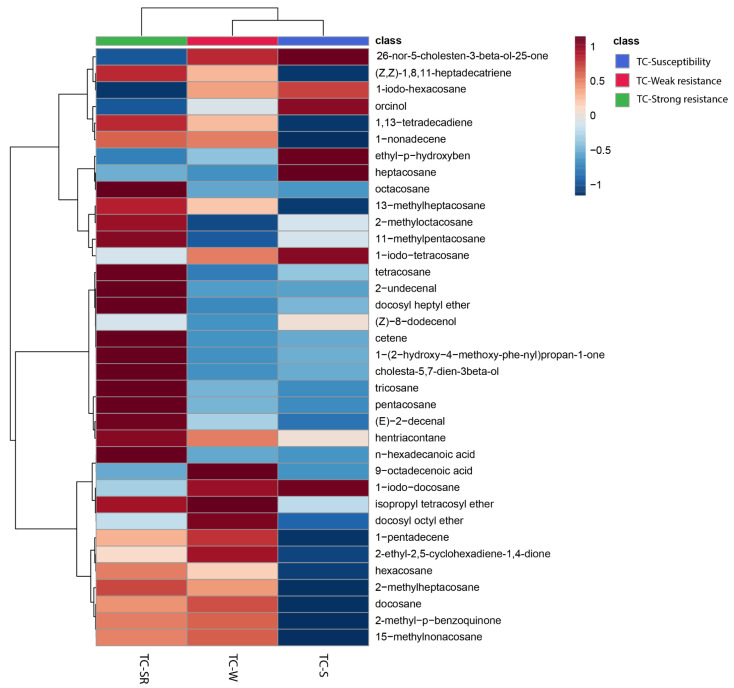
Heatmap showing the changes of abundance values normalized (with *p* < 0.05; calculated via a one-way ANOVA) to the metabolites that are significantly influenced by different PH_3_ resistance levels.

To enhance the visualization of the similarities and differences in the RA of metabolites in the TC-S, TC-W, and TC-SR groups, a heatmap was constructed to cluster the identified metabolites based on the similarity of their RA changes. As depicted in Figure 3, the 29 metabolites subjected to hierarchical clustering across the three groups demonstrated varied regulatory directions. Each cell within the heatmap signifies the mean RA of a metabolite, with the color gradient from blue to red indicating the downregulation to upregulation of metabolites, respectively. Notably, approximately half of the metabolites in the TC-W and TC-SR groups exhibited upregulation, whereas the majority in the TC-S group were downregulated. The heatmap thus provides a clear and intuitive representation of the distinct metabolite patterns among adult *T. castaneum* strains with varying degrees of PH_3_ sensitivity (susceptibility, weak PH_3_ resistance, and strong PH_3_ resistance).

### 2.3. Statistical Analysis and Differentially Regulated Metabolites

**Figure 4:**
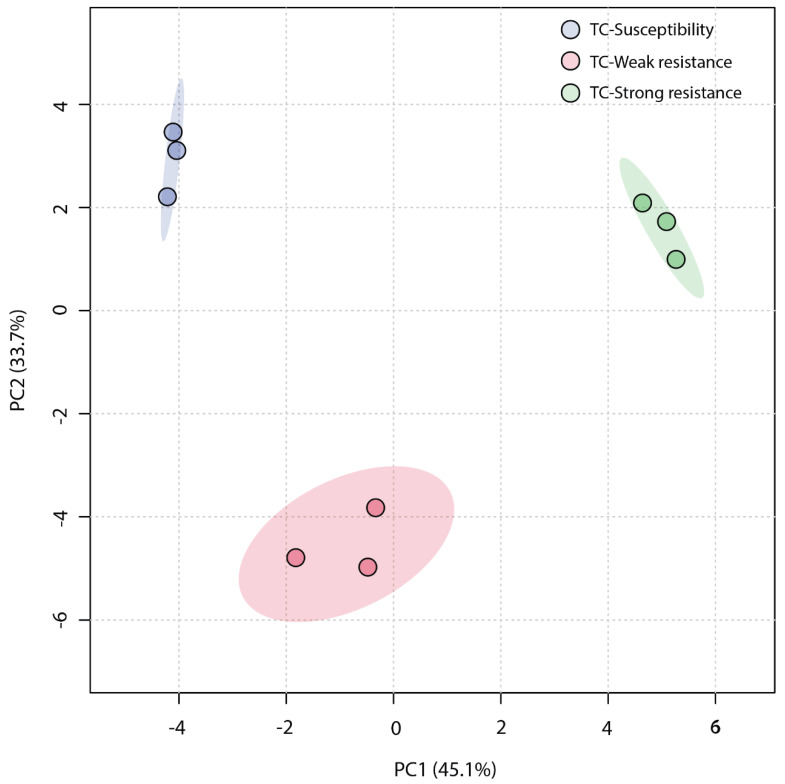
PCA score plot of metabolic profiles of *T. castaneum* adults from the TC-S, TC-W, and TC-SR groups.

**Figure 5:**
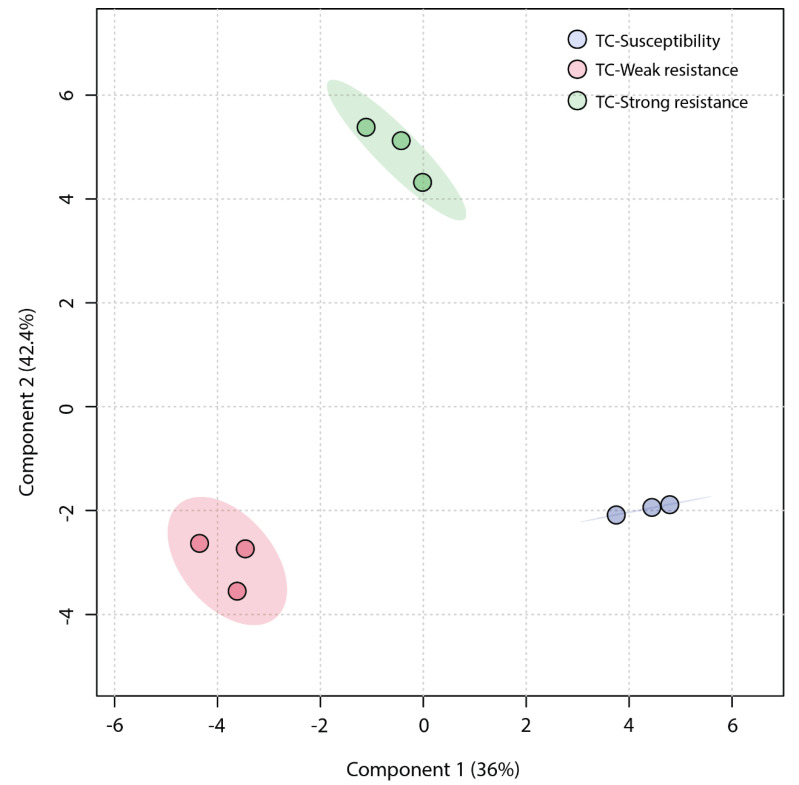
PLS-DA score plot of metabolic profiles of *T. castaneum* adults from the TC-S, TC-W, and TC-SR groups.

An unsupervised Principal Component Analysis (PCA) was employed to analyze the similarity among the three sample groups. The plot of the PCA scores (Figure 4) revealed that the first principal component (PC1) accounted for a significant proportion (45.1%) of the total variance in the original data, and the second principal component (PC2) explained 33.7%. The TC-W, TC-SR, and TC-S groups were clearly distinguished on the PC1 axis, with no overlap in the 95% confidence interval. On the PC2 axis, both TC-S and TC-SR were distinctly separated from the TC-W group. However, a clear separation between TC-S and TC-SR was not observed on this axis, potentially attributable to less significant inter-group differences. The Partial Least Squares Discriminant Analysis (PLS-DA) model, a supervised method, has greater distinguishing power compared to PCA models. As shown in Figure 5 (the score plot of the PLS-DA model), the inter-group distance of TC-W, TC-SR, and TC-S is larger, more clearly reflecting the differences among the three groups; moreover, the three groups demonstrated better clustering, indicating that PLS-DA successfully minimized the influence of intra-group variability. The above findings suggest that the emergence of PH_3_ resistance has significantly influenced the metabolic profiles of the *T. castaneum* adults, and this influence is strongly correlated with the level of resistance. The long-term domestication of PH_3_ has imposed an adaptive cost on the *T. castaneum*, and the changes in the content and composition of these metabolites may be the dynamic response to this adaptive cost.

**Figure 6:**
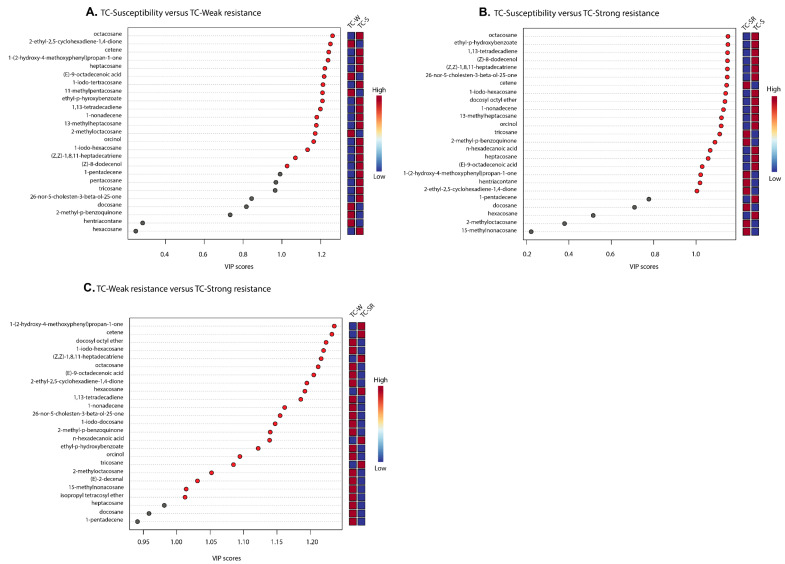
A variable importance plot showing the contribution of each metabolite to the first component (ranked based on VIP scores). (**A**) TC-S vs. TC-W, (**B**) TC-S vs. TC-SR and (**C**) TC-W vs. TC-SR.

“Differential metabolites” are defined as substances that are identified among different samples but show significant variations in respective concentrations [26]. The Variable Importance in Projection (VIP) value of the PLS-DA model (threshold >1), the *p*-value of the Student’s *t*-test (threshold <0.05), and the Fold Change (FC) value (threshold > 0.5) were utilized as criteria to identify differential metabolites between the TC-S, TC-W, and TC-SR groups [27,28]. In the comparisons of TC-S versus TC-M, TC-S versus TC-SR, and TC-M versus TC-SR, a total of 14, 14, and 17 differentially regulated metabolites were identified, respectively (Figure 6A–C). Detailed information regarding the identification of these metabolites is listed in Table 3. It can be seen that there are three, two, and nine key differentially regulated metabolites that have the potential to serve as biomarkers for detecting the PH_3_ resistance levels of *T. castaneum* adults.

**Figure 7:**
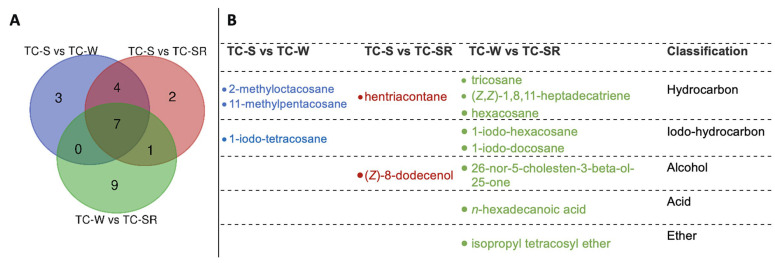
The amount (**A**) and chemical names (**B**) of specific differentially regulated metabolites of *T. castaneum* (Herbst) adults of TC-S versus TC-W, TC-S versus TC-SR, and TC-W versus TC-SR.

**Figure 8:**
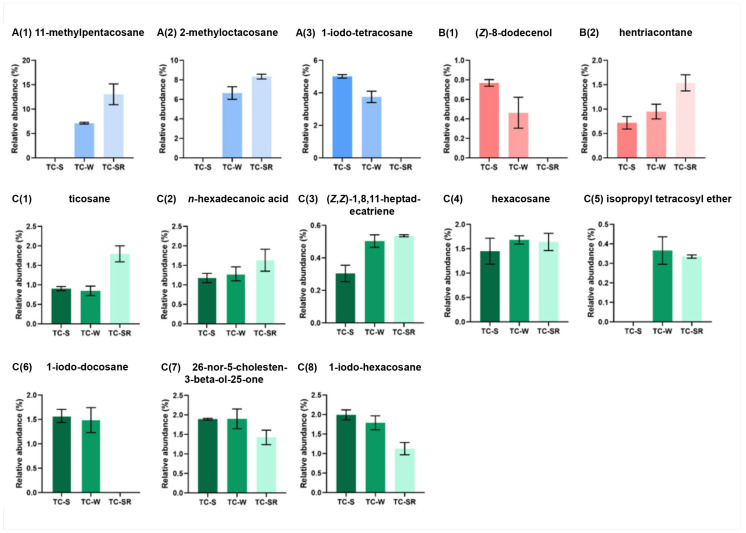
Specific differentially regulated metabolites RA from TC-S, TC-W and TC-SR groups of *T. castaneum* adults. **A(1)** 11-methylpentacosane, **A(2)** 2-methyl-octacosane, **A(3)** 1-iodo-tetracosane in TC-S vs. TC-W group; **B(1)** (*Z*)-8-dodecenol, **B(2)** hentriacontane in TC-S vs. TC-SR group; **C(1)** ticosane, **C(2)** *n*-hexadecanoic acid, **C(3)** (*Z*,*Z*)-1,8,11-heptadecatriene, **C(4)** hexacosane, **C(5)** isopropyl tetracosyl ether, **C(6)** 1-iodo-docosane, **C(7)** 26-nor-5-cholesten-3-beta-ol-25-one, **C(8)** 1-iodo-hexacosane in TC-W vs. TC-SR group.

These differentially regulated metabolites can be primarily categorized into several classes: hydrocarbons, iodo-hydrocarbons, alcohols, acids, and ethers (Figure 7 and Figure 8). Notably, among these three groups of differential metabolites, hydrocarbons constitute the majority, followed by iodo-hydrocarbons. Tricosane, hexacosane, 11-methylheptacosane, 2-methyloctacosane, 1,8,11-heptadecatriene (*Z*,*Z*)-, and hentriacontane have been identified as cuticular metabolites in *T. castaneum* [29], and their concentrations have been found to correlate positively with the level of PH_3_ resistance in *T. castaneum*. Research conducted by Alnajim et al. has previously reported that the levels of cuticular hydrocarbons in PH_3_-resistant strains is much higher than in sensitive strains [17]. This result aligns with the findings of the present study. The correlation may be attributed to the role of cuticular lipid metabolism in the development of insect PH_3_ resistance, leading to a significant accumulation of hydrocarbons in the cuticle. Consequently, this accumulation results in a pronounced reduction in the permeability of the insect cuticle, thereby enhancing resistance to PH_3_ in the insect [29]. Iodo-hydrocarbon compounds—e.g., (*Z*)-8-dodecenoland 26-nor-5-cholesten-3-beta-ol-25-one—are substances typically related to the synthesis of insect sex pheromones. In this study, a reduction in the RA of five distinct compounds—(*Z*)-8-dodecenol; 26-nor-5-cholesten-3-beta-ol-25-one; 1-iodo-tetracosane; 1-iodo-hexacosane and 1-iodo-docosane—was observed. This decrease may be linked to the potential fitness cost associated with enhanced PH_3_ resistance, resulting in the inhibition of sex pheromone production [30,31,32]. This finding strongly supports the results of a study conducted by Ridley et al. in which the reproductive capability of highly resistant *T. castaneum* was demonstrated to be significantly diminished in comparison to sensitive strains [33]. Fatty acids function as vital “fuel molecules” within biological organisms, facilitating ATP synthesis and enabling energy storage through esterification reactions. *N*-hexadecanoic acid is among the most prevalent fatty acids in organisms [34]. It plays a crucial role as an energy source in lipid metabolism and acts as a significant signaling molecule for assessing lipid content [35]. According to research by Schlipalius et al., the genes *rph1* and *rph2* can enhance PH_3_ toxicity by promoting lipid peroxidation and inflicting damage to fatty acids present on biological membranes [36]. Conversely, in PH_3_-resistant insects with *rph1* or *rph2* alleles, the sensitivity of the biological membrane to reactive oxygen species (ROS) is reduced, leading to a decrease in the consumption of fatty acids [29,37]. This finding further confirms the results of this study, suggesting that *n*-hexadecanoic acid can be employed as a biomarker to detect the resistance levels of *T. castaneum* to PH_3_. The content of the ether compound isopropyl tetracosyl ether exhibited significant variation but did not show a correlation with the PH_3_ resistance level in *T. castaneum*. This absence of correlation might be attributed to the individual differences among the test insects or the technique employed in metabolite extraction (DI-SPME). Recently, this type of substance has been detected by some researchers in studies exploring the impact of PH_3_ resistance on insect metabolic profiles, but detailed explanations have not been provided [17]. Consequently, the role of ether metabolites in insect PH_3_ resistance remains a ‘black box’, representing an intriguing direction for our subsequent research.

## 3. Materials and Methods

### 3.1. The Insect Culture

The Chinese Academy of Inspection and Quarantine (CAIQ) provided both susceptible and resistant *T. castaneum* adults (TC-S, TC-W, and TC-SR), which were initially collected from Wuhan (WH) in Hubei Province, Qihe (QH) in Shandong Province, and Zibo (ZB) in Shandong Province. To rear narrow-aged insects, a cohort of 1000 adult insects was incubated with 300 g of food consisting of wheat flour and yeast in a 9:1 ratio in a 4 L glass jar sealed with a meshed lid. After a 3-day spawning period, the adult insects were removed, and the remaining cultural medium containing eggs was incubated at 25 ± 2 °C and 65 ± 5% relative humidity. Emerging narrow-aged adults were subsequently transferred to a new jar with food to keep them at the same developmental stage. The insects utilized in the experiments were approximately one month old.

### 3.2. Fumigation Bioassay

The PH_3_ gas was generated from 56% aluminum phosphide tablets that had been immersed in a 5% aqueous solution of sulfuric acid. The concentration of this source gas was analyzed using a gas chromatograph (GC6890, Agilent Technology Co. Ltd., Santa Clara, CA, USA) equipped with a flame photometric detector (GC-FPD), with separation occurring in an Agilent J&W GS-GasPro column.

Fumigation bioassays with PH_3_ were conducted on *T. castaneum* adults inside 6-L desiccators, serving as fumigation chambers, at a controlled temperature of 25 ± 2 °C and 60 ± 5% RH. Plastic jars (40 mm inner diameter × 60 mm height) were utilized as containers for the test insects. Each chamber was assigned three jars, with each jar containing 30 *T. castaneum* adults. PH_3_ concentrations were selected within the ranges of 0.01–0.1 mg/L, 0.2–2.0 mg/L, and 1.0–10.0 mg/L for the TC-S, TC-W, and TC-SR strains, respectively, with approximately 5 to 8 concentrations chosen for each. Following a 20 h fumigation period, the desiccators were aerated for 2 h, after which the treated insects were removed and maintained at 25 ± 2 °C and 60 ± 5% RH. Mortality was assessed 72 h after fumigation.

### 3.3. Sample Preparation and Extraction Using DI-SPME

Before the extraction process, all insects used in this study were subjected to a cleaning process to ensure that their bodies were without feed residues. They were first placed on wet tissue and allowed to walk for 10 min, then transferred to dry tissue for an additional 10 min. For sample preparation, the insects were transferred into a 2 mL BeadBug™ microtube containing 1.6 mL of HPLC-grade acetonitrile (≥99.9%, Xilong Chemical Co. Ltd., Guangzhou, China) and 0.5 mm Silica glass beads and were homogenized at 4000 rpm for 1 min using a Beadbug homogenizer. Subsequently, the samples were centrifuged at 25 °C and 25,000× *g* for 3 min using a centrifuge (5417R, Eppendorf, Hamburg, Germany). The supernatant (1.3 mL) was transferred to a brown chromatography vial (Supelco, Darmstadt, Germany) with septa. The SPME fiber with a 50/30 µm Carboxen/DVB/PDMS coating (Sigma-Aldrich, Bellefonte, PA, USA) was inserted into the samples for extraction and conditioned for 1 h at 25 ± 1 °C. Then, GC-MS analysis was performed on the DI-SPME with a desorption time of 15 min. The samples were analyzed in biological triplicates.

### 3.4. Gas Chromatography-Mass Spectrometry (GC-MS) Conditions

The analysis was conducted using an Agilent 8890 GC (Agilent Technology Co. Ltd., Santa Clara, CA, USA), equipped with an HP-5MS capillary column (30 m × 0.25 mm, 0.25 μm; Agilent J&W Scientific) and coupled with an Agilent 5977B mass-selective detector (MSD, Agilent Technology Co. Ltd., Santa Clara, CA, USA). The carrier gas used was 99.999% purified helium, which was used at a constant flow rate of 1 mL/min. The injector temperature of the GC-MS was maintained at 270 °C. The initial temperature of the oven was set to 60 °C for 2 min; the temperature was then increased at 7 °C/min to 200 °C, 5 °C/min to 300 °C, and finally increased to 320 °C at 50 °C/min and maintained for 3 min. The ion source temperature was 230 °C, and the MSD transfer line temperature was 280 °C. Electron impact ionization (70 eV) was carried out at full-scan mode, ranging from 30 to 550 atomic mass units (amu), with a solvent delay time of 5 min. The total running time was 50.4 min.

### 3.5. Statistical Analyses

The GC-MS raw data were initially processed using Agilent MassHunter Qualitative Analysis Software (Version 10.0). Compounds were identified via comparison with the National Institute of Standards and Technology (NIST) and Wiley Registry ^®^ of Mass Spectral Data. The identification of these compounds was further supported by the retention index provided by the NIST Chemical Web Book, along with Kovat’s retention index.

Within the R software platform, the XCMS package was utilized to extract and pre-process the characteristic data from GC-MS. The edited data matrix was then imported into SIMCA software (Version 14.0) for multivariate statistical analyses, including Principal Component Analysis (PCA) and Partial Least Squares Discriminant Analysis (PLS-DA). To assess the statistical significance between groups, a one-way Analysis of Variance (ANOVA), followed by the Fisher’s least significant difference (LSD) test, was employed, and all analyses were conducted using SPSS Statistic 26.0. Additionally, unless otherwise specified, the significance level for evaluating differences was set at 0.05.

## 4. Conclusions

In this investigation, the DI-SPME technique was utilized to isolate volatile organic compounds from acetonitrile extracts of *T. castaneum* with three different levels of PH_3_ resistance. Our subsequent analysis carried out by employing GC-MS revealed differences in the metabolic profiles across these different PH_3_ resistance levels, with a number of differentially regulated metabolites showing trends correlated with PH_3_ resistance. These variations could be principally attributed to changes in the hydrocarbons and iodo-hydrocarbons within the PH_3_-resistant insects. Moreover, this study demonstrated that DI-SPME-GCMS is an efficient detection method that has the potential to distinguish insects at different PH_3_ resistance levels, allowing for the distinction of different PH_3_-resistant strains based on their unique chemical signatures. Overall, these insights improve our understanding of the underlying mechanisms of insect PH_3_ resistance from a metabolites standpoint and could form the basis for the development of innovative diagnostic methods for insect PH_3_ resistance analysis.

## Figures and Tables

**Table 1:** Probit mortality response of adults of *T. castaneum* to PH_3_ at 25 ± 2 °C and 55 ± 5% RH.

Strain	^1^ N	Slope ± SE	LC50 (mg/L) (95% ^2^ FL)	LC99 (mg/L)	Heterogeneity Factor	^3^ df	^4^ G -Factor	^5^ Mean Deviance Ratio	^6^ RR (^7^ CL)	Classification
Wuhan (TC-S)	1050	1.667 ± 0.242	0.011 (0.003, 0.014)	0.223	0.520	16	0.063	487.084 (*p* < 0.001)	-	Susceptibility
Qihe (TC-W)	900	2.037 ± 0.207	0.508 (0.443, 0.570)	7.049	0.401	16	0.284	676.357 (*p* < 0.001)	46.194 (40.298, 54.267)	Weak
Zibo (TC-SR)	900	9.084 ± 0.673	3.114 (2.988, 3.234)	5.615	0.570	16	0.027	585.656 (*p* < 0.001)	283.050 (271.678, 294.072)	Strong

^1^ N: Total number of insects used for the bioassay. ^2^ FL: Fiducial limit. ^3^ df: degrees of freedom. ^4^ G-factor = [t2 × V(b)/b2]m where t = student’s t with error degrees of freedom, V(b) is the slope variance estimate given in the variance-covariance matrix, and b is the slope estimate. G-values less than 0.5 suggest that the value of the mean is within the limit at 95% probability. ^5^ Fisher’s probability value is highly significant (<0.001). ^6^ Resistance ratio (RR): LC50 of resistant strain/LC50 of susceptible strain.^7^ CL: Confidence interval.

**Table 2:** Profile of metabolites produced from *T. castaneum* with different levels of PH_3_ resistance.

Metabolites	^1^ RT	^2^ RI (*Exp*)	^3^ RI (*Lit*)	RA (%) ± ^5^ SD			*p*-Value	CAS
				TC-S	TC-W	TC-SR		
2-methyl-*p*-benzoquinone	6.961	1042	1018	0.18 ± 0.02 ^b^	0.47 ± 0.02 ^a^	0.49 ± 0.02 ^a^	0.0000118	553-97-9
2-ethyl-2,5-cyclohexadiene-1,4-dione	8.915	1129	1215	0.3 ± 0.01 ^c^	0.69 ± 0.01 ^a^	0.54 ± 0.05 ^b^	0.0000250	4754-26-1
(*E*)-2-decenal	12.137	1225	1263	^4^ N.D. ^c^	0.3 ± 0.01 ^b^	0.38 ± 0.05 ^a^	0.0030101	3913-81-3
2-undecenal	13.708	1363	1367	N.D. ^c^	0.29 ± 0.04 ^b^	0.41 ± 0.05 ^a^	0.0041508	2463-77-6
orcinol	14.108	1421	1374	0.95 ± 0.16 ^a^	0.58 ± 0.10 ^b^	0.41 ± 0.06 ^b^	0.0074732	504-15-4
ethyl-*p*-hydroxybenzoate	15.254	1502	1438	1.92 ± 0.09 ^a^	1.23 ± 0.11 ^b^	1.17 ± 0.15 ^b^	0.0050822	2349-70-4
(*Z*)-8-dodecenol	16.104	1508	1468	0.77 ± 0.03 ^a^	0.46 ± 0.16 ^b^	N.D. ^c^	0.0020082	40642-40-8
1-pentadecene	16.461	1527	1515	15.93 ± 0.66 ^a^	15.56 ± 1.71 ^a^	15.63 ± 3.01 ^a^	0.1367729	13360-61-7
1-(2-hydroxy-4-methoxyphenyl)propan-1-one	17.338	1653	1538 *	0.96 ± 0.04 ^b^	0.75 ± 0.04 ^b^	1.33 ± 0.20 ^a^	0.0005612	6270-44-6
cetene	18.098	1612	1592	0.50 ± 0.03 ^b^	0.39 ± 0.01 ^c^	0.83 ± 0.08 ^a^	0.0000209	629-73-2
(*Z*,*Z*)-1,8,11-heptadecatriene	19.271	1620	1665	0.28 ± 0.04 ^a^	0.50 ± 0.04 ^b^	0.54 ± 0.01 ^b^	0.0014243	56134-03-3
1,13-tetradecadiene	19.41	1709	1867	4.81 ± 0.46 ^b^	5.63 ± 0.41 ^b^	7.32 ± 0.40 ^a^	0.032606	21964-49-8
1-nonadecene	19.756	1717	1892	5.87 ± 0.31 ^b^	5.86 ± 0.57 ^b^	7.14 ± 0.32 ^a^	0.7961594	18435-45-5
*n*-hexadecanoic acid	23.809	2016	1968	1.13 ± 0.05 ^a^	1.28 ± 0.18 ^a^	1.65 ± 0.28 ^a^	0.0041685	57-10-3
(*E*)-9-octadecenoic acid	26.506	2154	2139	0.81 ± 0.02 ^b^	1.62 ± 0.31 ^a^	0.77 ± 0.03 ^b^	0.0020028	112-79-8
docosane	31.904	2402	2200	0.16 ± 0.01 ^b^	0.46 ± 0.08 ^a^	0.43 ± 0.02 ^a^	0.015176	629-97-0
tricosane	32.981	2443	2300	0.90 ± 0.05 ^b^	0.85 ± 0.12 ^b^	1.68 ± 0.02 ^a^	0.00000754	638-67-5
tetracosane	33.358	2505	2400	N.D. ^c^	0.27 ± 0.01 ^b^	0.44 ± 0.00 ^a^	0.0000532	646-31-1
pentacosane	33.496	2507	2500	0.37 ± 0.03 ^a^	0.33 ± 0.06 ^a^	0.47 ± 0.01 ^a^	0.0016095	629-99-2
hexacosane	33.869	2703	2600	1.45 ± 0.27 ^a^	1.68 ± 0.08 ^a^	1.58 ± 0.21 ^a^	0.014387	630-01-3
heptacosane	34.275	2711	2700	0.78 ± 0.11 ^a^	0.45 ± 0.04 ^b^	0.48 ± 0.06 ^b^	0.010739	593-49-7
isopropyl tetracosyl ether	34.458	2720	2724	N.D. ^b^	0.37 ± 0.07 ^a^	0.33 ± 0.01 ^a^	0.039729	^6^ N.A.
1-iodo-docosane	34.675	2735	2730	1.57 ± 0.13 ^b^	1.38 ± 0.21 ^b^	N.D. ^a^	0.0813	1000406-31-9
13-methylheptacosane	35.376	2805	2731	0.22 ± 0.02 ^b^	0.42 ± 0.05 ^a^	0.45 ± 0.01 ^a^	0.2679168	15689-72-2
11-methylpentacosane	35.487	2809	2734	N.D. ^c^	7.12 ± 0.17 ^b^	13.03 ± 2.13 ^a^	0.0094031	15689-71-1
2-methylheptacosane	35.561	2829	2762	1.40 ± 0.23 ^a^	1.70 ± 0.21 ^a^	1.87 ± 0.34 ^a^	0.0098973	1561-00-8
octacosane	35.756	2856	2800	3.12 ± 0.23 ^b^	3.11 ± 0.38 ^b^	7.36 ± 0.09 ^a^	0.00000444	630-02-4
2-methyloctacosane	35.958	2870	2859	N.D. ^c^	6.65 ± 0.65 ^b^	8.34 ± 0.25 ^a^	0.361	1560-88-9
15-methylnonacosane	36.128	2804	2923	1.76 ± 0.08 ^b^	2.25 ± 0.28 ^a^	2.18 ± 0.14 ^ab^	0.0012749	65820-60-2
1-iodo-tetracosane	36.818	2873	2942	5.01 ± 0.11 ^a^	3.75 ± 0.35 ^b^	N.D. ^c^	0.104	1000406-32-0
docosyl heptyl ether	37.238	2910	2966	N.D. ^a^	0.27 ± 0.05 ^b^	0.39 ± 0.02 ^a^	0.0064187	N.A.
docosyl octyl ether	37.983	3046	3056	1.2 ± 0.04 ^b^	1.50 ± 0.11 ^a^	1.23 ± 0.14 ^ab^	0.0005646	N.A.
hentriacontane	38.315	3003	3100	0.72 ± 0.13 ^b^	0.95 ± 0.15 ^b^	1.44 ± 0.04 ^a^	0.0000762	630-04-6
26-nor-5-cholesten-3-beta-ol-25-one	38.540	3022	3131	1.89 ± 0.02 ^a^	1.90 ± 0.25 ^a^	1.33 ± 0.12 ^b^	0.0025098	7494-34-0
1-iodo-hexacosane	40.449	3172	3147	1.99 ± 0.13 ^a^	1.79 ± 0.18 ^a^	1.05 ± 0.14 ^b^	0.0001359	52644-81-2
cholesta-5,7-dien-3beta-ol	40.958	3201	3158	N.D. ^c^	0.19 ± 0.03 ^b^	0.29 ± 0.03 ^a^	0.0647143	434-16-2

^1^ RT: Retention time. ^2^ RI (*Exp*): Retention Index (determined by calculated using *n*-alkane standard C7–C40). ^3^ RI (*Lit*): Standard of Retention Index from NIST Library. ^4^ N.D.: Not detected. ^5^ SD: Standard deviation. ^6^ N.A.: Not available. * Estimated non-polar retention index (*n*-alkane scale NIST). Values represent the means of three replicates, and values within the same row with different superscript letters (a, b and c) indicate significant differences between the TC-S, TC-W, and TC-SR groups at *p* < 0.05.

**Table 3:** Differential metabolites in TC-S versus TC-W, TC-S versus TC-SR, and TC-W versus TC-SR comparisons (VIP > 1, *p* < 0.05 and |Log2(FC)| > 0.5).

TC-S versus TC-W	*p*-Value	log2(FC)	TC-S versus TC-SR	*p*-Value	log2(FC)	TC-W versus TC-SR	*p*-Value	log2(FC)
octacosane	0.0000309	1.5416	octacosane	0.00000236	1.5416	cetene	0.000143	−0.6049
2-ethyl-2,5-cyclohexadiene-1,4-dione	0.000264	0.90981	ethyl-*p*-hydroxybenzoate	0.00000355	−0.93771	docosyl octyl ether	0.00042	0.79877
cetene	0.000599	−0.674	1,13-tetradecadiene	0.0000138	0.67309	1-iodo-hexacosane	0.000583	−1.248
1-(2-hydroxy-4-methoxyphenyl)propan-1-one	0.000784	−0.66323	(*Z*)-8-dodecenol	0.0000174	−0.7814	(*Z*,*Z*)-1,8,11-heptadecatriene	0.000764	1.3119
heptacosane	0.001893	−1.1068	cetene	0.0000687	−0.674	octacosane	0.001025	0.51017
(*E*)-9-octadecenoic acid	0.002226	0.67888	1-nonadecene	0.000585	0.5782	(*E*)-9-octadecenoic acid	0.001478	1.5648
1-iodo-tetracosane	0.002793	−0.71705	13-methylheptacosane	0.001161	0.99779	2-ethyl-2,5-cyclohexadiene-1,4-dione	0.002354	0.88627
11-methylpentacosane	0.003041	1.1804	orcinol	0.001259	−1.0116	hexacosane	0.002634	0.61333
ethyl-*p*-hydroxybenzoate	0.00307	−0.93771	2-methyl-*p*-benzoquinone	0.004376	0.78326	1,13-tetradecadiene	0.003282	0.72677
1,13-tetradecadiene	0.004294	0.67309	heptacosane	0.009924	−1.1068	26-nor-5-cholesten-3-beta-ol-25-one	0.007607	0.99856
1-nonadecene	0.006906	0.5782	(*E*)-9-octadecenoic acid	0.016531	0.67888	1-iodo-docosane	0.00889	−1.6578
13-methylheptacosane	0.007303	0.99779	1-(2-hydroxy-4-methoxyphenyl)propan-1-one	0.018477	−0.66323	2-methyl-*p*-benzoquinone	0.010275	0.5094
2-methyloctacosane	0.008263	0.62565	hentriacontane	0.019355	0.097868	*n*-hexadecanoic acid	0.01046	1.044
orcinol	0.009666	−1.0116	2-ethyl-2,5-cyclohexadiene-1,4-dione	0.023668	0.90981	ethyl-*p*-hydroxybenzoate	0.014055	0.5571
						orcinol	0.020883	0.96149
						tricosane	0.023572	0.5086
						isopropyl tetracosyl ether	0.050881	−0.68621

## Data Availability

All data are contained within the article.

## Supplementary Materials

The following supporting information can be downloaded at: https://www.mdpi.com/article/10.3390/molecules28237721/s1, Figure S1: The chromatogram of each group.
