# Supplementary material for: Comparative Analysis of the Metabolic Profiles of Strains of Tribolium castaneum (Herbst) Adults with Different Levels of Phosphine Resistance Based on Direct Immersion Solid-Phase Microextraction and Gas Chromatography-Mass Spectrometry
Source: Molecules. 2023 Nov 22;28(23):7721. doi: 10.3390/molecules28237721 (PMC10707947; doi:10.3390/molecules28237721)
Supplement: Supplementary file 1 [file molecules-28-07721-s001.zip › molecules-2587398-supplementary.pdf]

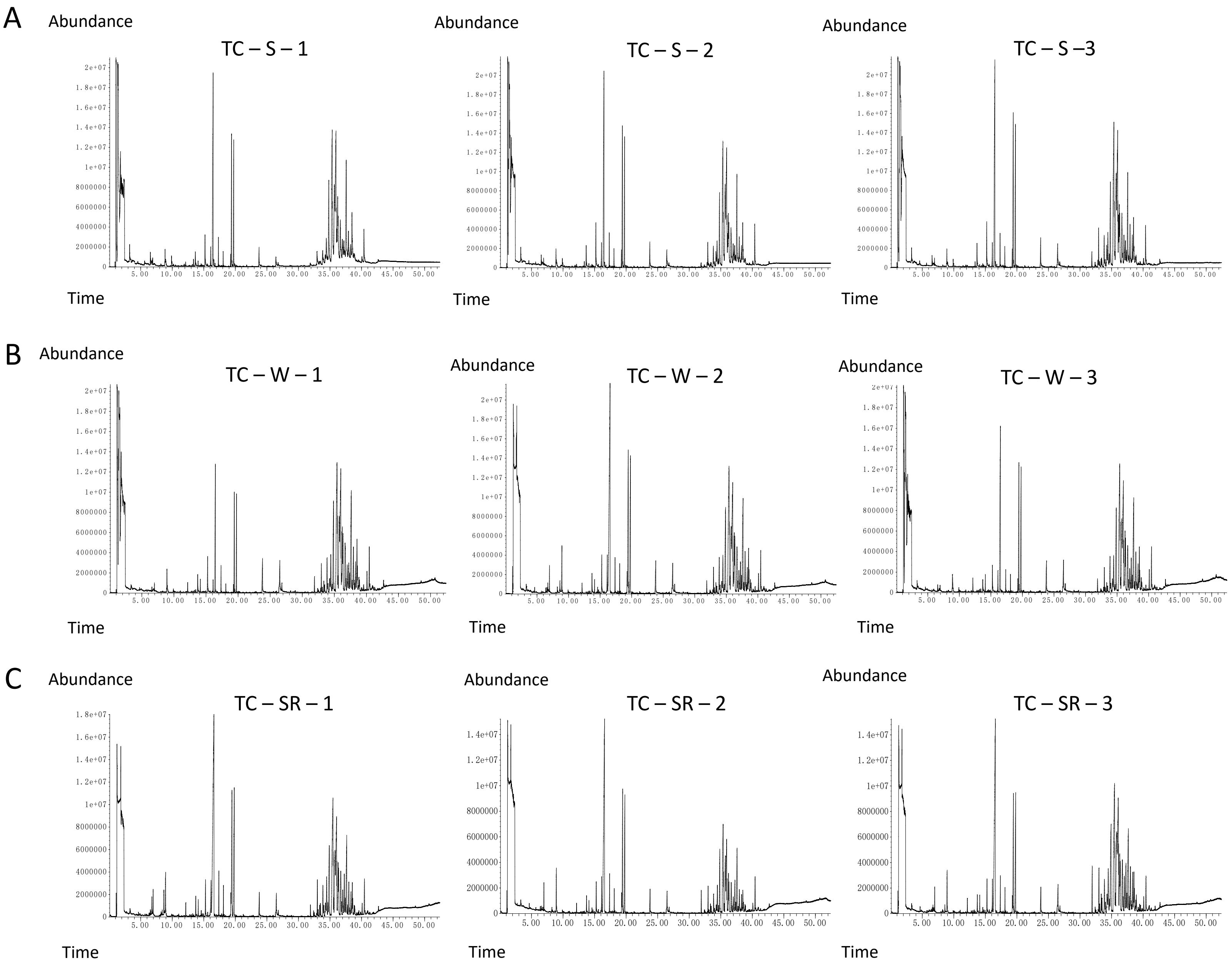
A
Abundance
TC – S – 1
Time
Abundance
TC – S – 2
Time
Abundance
TC – S –3
Time
B
Abundance
TC – W – 1
Time
Abundance
TC – W – 2
Time
Abundance
TC – W – 3
Time
C
Abundance
TC – SR – 1
Time
Abundance
TC – SR – 2
Time
Abundance
TC – SR – 3
Time

Figure S1. A: Chromatograms from each of the TC–S groups (TC–S– 1, TC–S–2 and TC–S–3); B: Chromatograms from each of the TC–W groups (TC–W–1, TC–W–2 and TC–W–3); C: Chromatograms from each of the TC–SR groups (TC–SR– 1, TC–SR–2 and TC–SR–3).
